# Structural and Functional Aspects of Targeting the Secreted Human Group IIA Phospholipase A_2_

**DOI:** 10.3390/molecules25194459

**Published:** 2020-09-28

**Authors:** Ryung Rae Kim, Zheng Chen, Timothy J. Mann, Karine Bastard, Kieran F. Scott, W. Bret Church

**Affiliations:** 1 School of Pharmacy, Faculty of Medicine and Health, University of Sydney, Sydney, NSW 2006, Australia; rkim2691@uni.sydney.edu.au (R.R.K.); zche6274@uni.sydney.edu.au (Z.C.); karine.bastard@sydney.edu.au (K.B.); 2 School of Medicine, Western Sydney University, Centre for Oncology, Education and Research Translation and The Ingham Institute, Liverpool, NSW 2170, Australia; 17432636@student.westernsydney.edu.au

**Keywords:** phospholipase A_2_, GIIA, inflammation, cancer, LY311727, LY315920, LY333013, varespladib, varespladib methyl, FLSYK, KH064, c2, *p*-Bromophenacyl bromide, BPB, arachidonic acid cascade, cyclooxygenase, COX

## Abstract

Human group IIA secretory phospholipase A_2_ (hGIIA) promotes the proliferation of cancer cells, making it a compelling therapeutic target, but it is also significant in other inflammatory conditions. Consequently, suitable inhibitors of hGIIA have always been sought. The activation of phospholipases A_2_ and the catalysis of glycerophospholipid substrates generally leads to the release of fatty acids such as arachidonic acid (AA) and lysophospholipid, which are then converted to mediator compounds, including prostaglandins, leukotrienes, and the platelet-activating factor. However, this ability of hGIIA to provide AA is not a complete explanation of its biological role in inflammation, as it has now been shown that it also exerts proinflammatory effects by a catalysis-independent mechanism. This mechanism is likely to be highly dependent on key specific molecular interactions, and the full mechanistic descriptions of this remain elusive. The current candidates for the protein partners that may mediate this catalysis-independent mechanism are also introduced in this review. A key discovery has been that selective inhibition of the catalysis-independent activity of hGIIA is achieved with cyclised derivatives of a pentapeptide, FLSYK, derived from the primary sequence of hGIIA. The effects of hGIIA on cell function appear to vary depending on the pathology studied, and so its mechanism of action is complex and context-dependent. This review is comprehensive and covers the most recent developments in the understanding of the many facets of hGIIA function and inhibition and the insight they provide into their clinical application for disease treatment. A cyclic analogue of FLSYK, c2, the most potent analogue known, has now been taken into clinical trials targeting advanced prostate cancer.

## 1. hGIIA and the Phospholipase A_2_ Superfamily

The phospholipases A_2_ (PLA_2_s) family are esterases that catalyse the hydrolysis of the *sn-2* position of glycerophospholipids to release free fatty acids and lysophospholipids. Depending on physical characteristics, biological location, calcium dependency, catalytic mechanism and substrates, these enzymes can be categorised into four broad classes: secreted (sPLA_2_), cytosolic (cPLA_2_), calciumin-dependent (iPLA_2_) and lipoprotein-associated (Lp-PLA_2_). Secretory phospholipases are some of the earliest identified enzymes and were isolated from snake venom in the late 19th century [1]. Secreted PLA_2_s (sPLA_2_s) are a group of PLA_2_ enzymes (Table 1) that may be secreted extracellularly, as the name suggests, but some members are also able to be internalised into the intracellular space.

**Figure 1:**
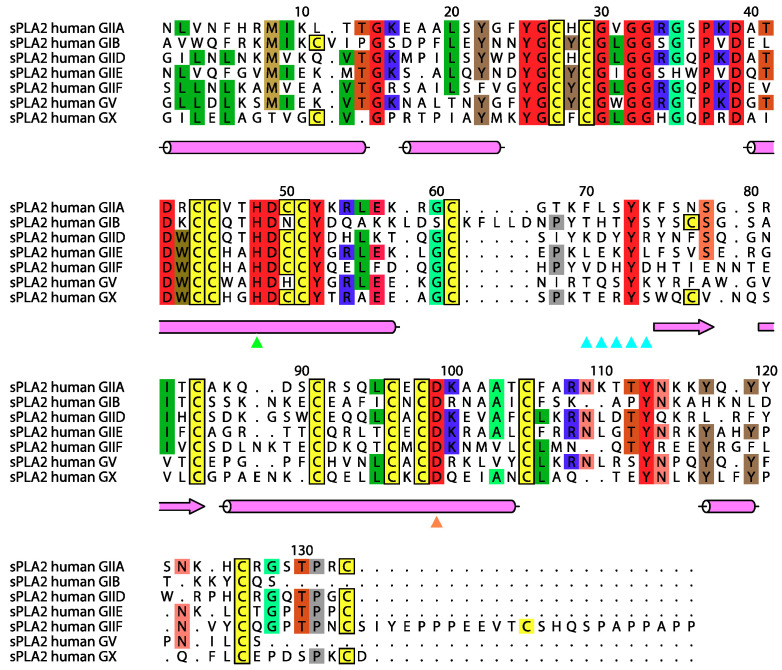
Sequence alignments of seven human secreted phospholipases A_2_. Overall the locations of high conservation of sequence arising from the active site, the calcium binding and disulfide bond are evident. Highlighting occurs when there is identity for at least 4 of the sequences, with complete conservation among these proteins shown in red, except that all Cys are highlighted as yellow. Cys known to be involved in disulfide bonds are boxed. The other highlighted amino acids occur for four or more identities and are coloured according to amino acid type. Below the sequences is the secondary structure as observed in hGIIA (cerise; α-helices as cylinders and β-strands as arrows), and below again are key locations in the sequence indicated with triangles: catalytic His (green), catalytic Asp (orange), calcium binding loop (cyan). The location of the FLSYK is given with cyan triangles. The numbering shown is that of Renetseder et al. 1985 [4], which is universally used for hGIIA in this paper, but not guaranteed for the other entries. Figure created with ALINE [5].

The enzymes have a low molecular weight compared to other PLA_2_s, ranging between 13 and 19 kDa, with the exception of the group III subtype, which has N- and C-terminal extensions, and possess at least six highly conserved disulfide bonds. The sPLA_2_ enzymes have an active site sequence (Asp-Xxx-Cys-Cys-Xxx-Xxx-His-Asp) and a calcium binding loop (Xxx-Cys-Gly-Xxx-Gly-Gly) that are highly conserved across the subtypes (Figure 1). The histidine in the active site in the sequence shown (His48) forms a catalytic dyad with an aspartate remote in the sequence (Asp99) and this is also conserved across the subtypes.

The nomenclature and history of the PLA_2_ superfamily is well described in a review article by Dennis et al. [1]. The first characterised sPLA_2_s were from snake venom and, depending on the pattern of the disulfide bonds, these were either designated as Type 1 (cobras) and Type 2 (rattlesnakes) [1]. The first mammalian enzyme (subtype IB) isolated was of porcine origin, and is a pancreatic digestive enzyme that had a similar disulfide bond pattern to that of the cobra venom subtype [6]. This was then followed by the isolation of the human IB subtype [7]. The next human sPLA_2_ characterised was first purified from the synovial fluids from arthritic patients attracting clinical interest. The cDNA was cloned independently by researchers at California Biotechnology Inc [8] and Biogen Research Corporation [9] and is designated subtype IIA, as the disulfide pattern resembled that of the rattlesnake venom enzyme [1,10,11]. The literature has referred to the human group IIA sPLA_2_ (hGIIA), as human non-pancreatic PLA_2_ or human synovial fluid PLA_2_. The main property that separates group I and II sPLA_2_s is a unique disulfide bond, in addition to the six common ones formed between Cys 11 and Cys 77 in the group I and Cys 50 to Cys 133 in the group II [12]. In addition, the group I sPLA_2_s have an insertion at the base of the active site helix (at position 62 in Figure 1) and the group II enzymes have a C-terminal insertion. Figure 1 also shows that hGIIA has high sequence similarity to other subtypes of sPLA_2_ as well as high degree of interspecies conservation.

## 2. Catalytic Mechanism of hGIIA

**Figure 2:**
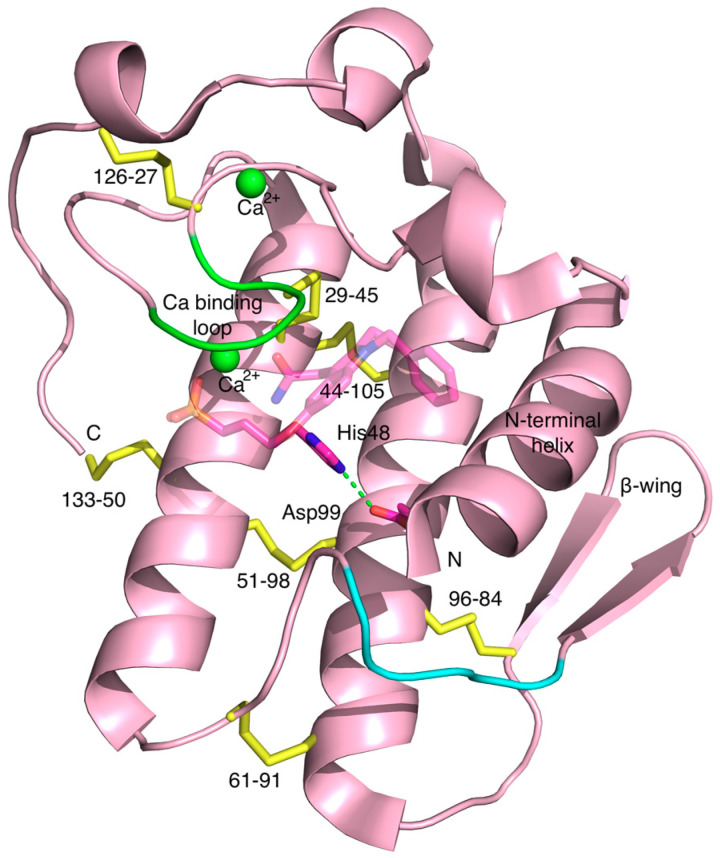
The three-dimensional structure of hGIIA in which the location of some of the functional and structural groups of hGIIA are depicted. The main chain is shown in cartoon style in cerise except for the cysteines in disulfide bonds (yellow), and the calcium binding loop (green) and calcium ions are also shown (green spheres). His48 and Asp99 with carbons in scarlet are the amino acids that constitute the catalytic dyad. The LY311727 inhibitor is also shown, with scarlet carbons and significantly transparent. Overall structure is native hGIIA (PDB ID 3U8B, 2.3 Å resolution [13]), and the LY311727 is superposed from PDB ID 3U8D (1.8 Å resolution) [13]. The region of the endogenous FLSYK sequence is shown in cyan on the cartoon. N and C represent the N- and C-termini, respectively. Figure created with PyMOL [14].

The highly conserved structural feature among the sPLA_2_ enzymes is the catalytic dyad utilised for substrate hydrolysis which consists of a histidine and an aspartate in close proximity to each other on examination of the three-dimensional structure (Figure 2). Because both are charged, with one being basic and the other being acidic, there is an electrostatic interaction between the carboxyl δ oxygen atom (O^δ1^) of the aspartate and the ε nitrogen atom of the histidine ring (N^ε2^). They closely resemble the classical catalytic triad in serine hydrolases that consists of serine, a basic and an acidic amino acid, but obviously lacks the serine that acts as a nucleophile with which it attacks the substrate initiating the hydrolysis. The sidechains of the dyad are shown in the context of the overall structure of hGIIA in Figure 2. It is deeply buried inside the protein at the floor of the active site cavity and found in close proximity to the calcium ion bound to the calcium binding loop.

**Figure 3:**
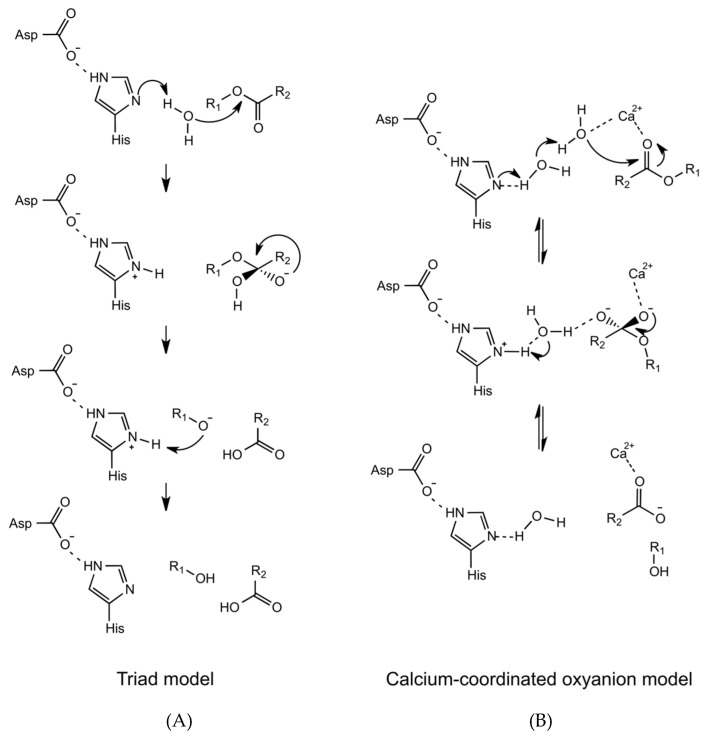
Two models of catalytic mechanism of hGIIA are depicted: (**A**) In the triad model, the water molecule becomes activated by the adjacent histidine N^δ1^ atom, and acts as a nucleophile to directly attack the carbonyl of the substrate; (**B**) In the calcium-coordinated oxyanion model, a second water molecule is linked to the N^δ1^ atom through the adjacent water molecule and also coordinated to the calcium ion. The water molecule and the calcium ion polarise the carbonyl group to initiate the cleavage, and the activation energy required is lower than the triad model. Adapted from Berg et al. [18].

In one of the earliest proposed catalytic mechanisms of hGIIA is the triad model (Figure 3), the water molecule bound to the N^δ1^ atom of the active site histidine was put forward to act as a nucleophile, in place of the serine of the serine hydrolases [15,16]. The catalytic mechanism may be referred to as originating from a dyad of histidine and aspartic acid, as the serine is absent from the triad of amino acids that are equivalent for the serine proteases. In the role of the nucleophile, it is then that the water molecule that becomes polarised by the N^δ1^ atom, and attacks the carbonyl group of the phospholipid substrate [15,17]. This is partially aided by the presence of the aspartate of the catalytic dyad, as the electrostatic interaction with the histidine N^ε2^ atom makes the N^δ1^ atom more electrophilic. Two calciums are commonly observed in the structures, and the calcium closest to the active site plays an essential role in this mechanism, as it interacts with the conserved aspartate Asp49 next to the active site histidine. It is coordinated by three carbonyl backbone oxygens from His28, Gly30 and Gly32, on the calcium binding loop, and both the carboxyl oxygens from the sidechain of Asp49. The calcium ion stabilises the intermediate by coordinating the carbonyl group of the phospholipid substrate and the negative charge from the phosphate oxygen. The glycines of the loop, of which there are an abundance, are able to achieve the polypeptide conformation that is not otherwise attainable.

In an alternative mechanism that was subsequently proposed (Figure 3B), a water molecule in addition to the one proposed is also involved in the catalysis [18,19]. In the crystal structure of hGIIA [19] a second water molecule is coordinated to the calcium ion cofactor and positioned 2.8 Å away the water molecule bound to N^δ1^ atom of the active site histidine. This water molecule indirectly interacts with the histidine N^δ1^ atom by forming a hydrogen bond with another water molecule which then makes a connection through to the N^δ1^ atom. Consequently, when the N^δ1^ atom becomes protonated the water molecule becomes polarised and may act as a nucleophile. The calcium ion plays an important role in this mechanism. The polarised water attacks the substrate carbonyl group to form an oxyanion, which coordinates the calcium ion. In comparison to the first mechanism, the involvement the second water molecule in the second mechanism lowers the activation energy of the required tetrahedral formation that protonates the N^δ1^ atom. In both mechanisms, the calcium ion acts as an important cofactor in the hydrolysis process, albeit for different reasons. Therefore, these mechanisms are consistent with the requirement of up to millimolar concentrations of calcium for the catalytic activity of hGIIA in experimental conditions [9]. The oxyanion mechanism is also more consistent, with lower activity of mutants with substituted His48 [20,21].

In order for the hydrolysis action to occur, the enzyme must initially bind with the phospholipid substrate. Generally, the phospholipid molecules are found in aggregated forms, such as micelles or bilayers, rather than as discrete molecules, and hence the binding to the substrate involves adsorption of the enzyme to the substrate surface. Two models of the kinetics have been suggested to describe how the enzyme behaves once it is adsorbed to the substrate surface [18,22,23]. In the ‘hopping mode’ model, the enzyme would bind to the substrate surface, hydrolyse only a single substrate molecule, and then detach from the surface before it finds the next substrate molecule to hydrolyse. In the ‘scooting mode’ model, the enzyme proceeds with multiple cycles of hydrolysis of the substrate molecules without desorbing from the substrate surface. In both mechanisms, it is essential for the free enzyme (E) to form a surface-bound form (E*) before it proceeds with classical Michaelis–Menten kinetics, as depicted in the simplified equation below.

(1) $$E\;\begin{array}{c} ⇌\\ K_{d} \end{array}{\;E}^{*}\;\begin{array}{c} ⇌\\ K_{M}^{*} \end{array}E^{*}S\;\begin{array}{c} \to \\ K_{s}^{*} \end{array}{\;E}^{*}P\;\begin{array}{c} ⇌\\ K_{P}^{*} \end{array}E^{*}+P\;$$

Indeed, the in vitro functional assays of hGIIA showed that the enzyme activity was significantly accelerated when the substrate concentration reached the critical micelle/bilayer concentration, as the enzyme in the free form is much less efficient at catalysis [24,25,26]. The enzyme kinetics of sPLA_2_s is heavily dependent on the ability of the enzyme to adsorb to the phospholipid surface (K_d_) but also on each individual phospholipid molecule to bind to the active site before the hydrolysis (K*_M_).

**Figure 4:**
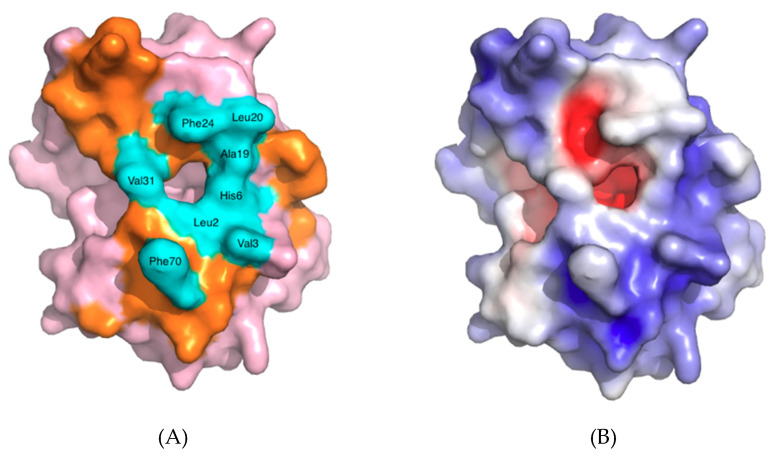
hGIIA shown with space-filling surface: (**A**) The amino acids highlighted in cyan, and identified, form the entrance to the active site, and play a role in interfacial binding by making direct contact with the substrate. The interfacial binding surface (i-face) of hGIIA outside of the amino acids at the entrance to the active site are highlighted in orange; (**B**) Depiction of the electrostatic charge. The high basicity of the protein at the surface is shown by the dominance of blue (positive), over the red of acidity (negative). Confirmation of the non-polar, hydrophobic entrance in orange in the left image is the coincident white of neutrality. The dominance of positive charge is clear. The view 180° to this (not shown) has less negative charge again, but does clearly have both a localised hydrophobic patch at the top in the current view and a localised very positive patch relative to the surface shown at the left hand side region near the C-terminus (behind the right hand side in the views given). Figure used PDB ID 3U8B [13] and created with PyMOL using the Adaptive Poisson-Boltzman Solver for the electrostatics [14]. The orientation is the same as used for Figure 2.

The part of sPLA_2_ proteins that is involved in direct binding to the phospholipid substrate surface forms a surface called the interfacial binding surface (i-face) [22,27]. These amino acids are mostly hydrophobic and exert strong hydrophobic interactions, but may also include some cationic residues that can form hydrogen bonds with anionic substances, as is the case for hGIIA. Within the i-face there are amino acids that form the entrance to the active site, and that also play an important role in interacting with the substrate or inhibitors (Figure 4A). These amino acids are mostly hydrophobic as well, but also form a relatively planar surface to the external surroundings. The entrance is where the single phospholipid molecule can enter and penetrate through to the active site where the catalytic dyad is present at the end of the cavity.

## 3. Substrate Specificity of hGIIA

In comparison with other human sPLA_2_ subgroups, such as group V and group X, or other mammalian GIIAs, hGIIA exhibits lower catalytic activity towards the zwitterionic phosphatidylcholine (PC) bilayer substrates [24,28,29,30]. The affinity of hGIIA towards PC is very low in comparison to those of the anionic class such as phosphatidic acid (PA), phosphatidylglycerol and phosphatidylserine (PS) [24,31,32]. For example, the catalytic activity of hGX towards the phosphocholine 2-oleoyl-1-pamlitoyl-sn-glyecro-3-phosphocholine (POPC) is 42 times higher than that of hGIIA, while towards the phosphoglyceride 2-oleoyl-1-pamlitoyl-sn-glyecro-3-glycero (POPG), it is 15 times lower than that of hGIIA [29]. Such selectivity towards anionic glycerophospholipids would appear to be associated with the unusually large proportion of basic amino acids that hGIIA contains relative to the other subtypes. There are 23 basic amino acids in hGIIA, constituting 18.5% of the total. and five of these are located around the i-face (Figure 4B). However, the argument against this hypothesis is evident in a study that used group III bee venom sPLA2 to create mutations on the five basic amino acids on the i-face to neutral-charged glutamine {Bollinger, 2004 #229]. This mutation caused only a relatively small 3-fold reduction in the enzyme activity towards the PS vesicles, suggesting that the interfacial binding is predominantly driven by the non-electrostatic forces. On the other hand, the charge-reversal mutation to glutamate significantly decreased the activity by 3000-fold [33], as the repulsive forces created by this mutation would deter binding of the anionic phospholipids to the protein.

The substrate specificity of hGIIA is also partially explained by the absence of a tryptophan on the i-face, as tryptophan is present on the i-face in several sPLA_2_s with relatively non-selective substrate specificity and significant catalytic activity towards the zwitterionic PC membranes. Some examples include the Indian cobra (*Naja naja*) venom sPLA_2_ at position 20 or other subgroups of human sPLA_2_, such as the group V subtype, which has Trp31. The amphiphilic indole moiety of the tryptophan would promote the penetration of the enzyme into the lipid interface of the phospholipid bilayer, thereby allowing the substrate access to the catalytic active site. In fact, the Val3Trp mutation introduced into hGIIA enhanced the activity towards unilamellar PC vesicles by over 250-fold [34]. Conversely, the mutation of Trp31 to alanine in the human group V subtype sPLA_2_ decreased the activity towards PC by 44-fold [28].

## 4. Biological Role of hGIIA

### 4.1. The Arachidonic Acid Cascade

**Figure 5:**
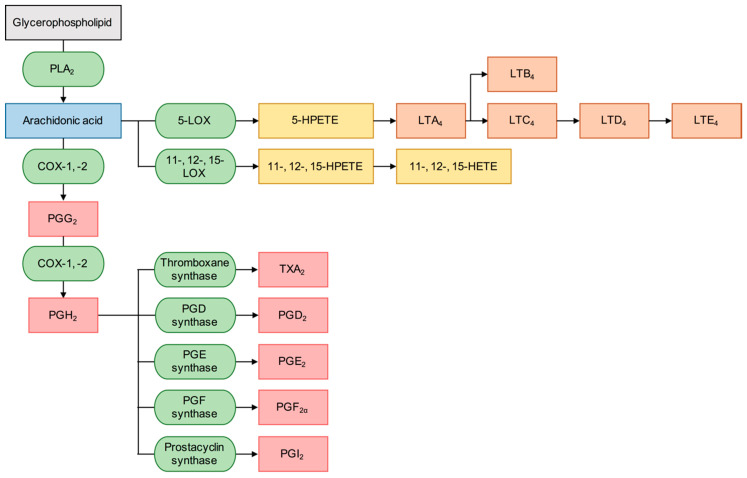
A simplified depiction of the arachidonic acid cascade where the green ellipses represent the enzymes and boxes represent the substrates and products associated with the pathway. The prostaglandins and thromboxane produced in the eicosanoid pathway are metabolites of cyclooxygenases (COXs) and highlighted in pink. The leukotrienes shown in orange are the metabolites of 5-lipoxygenase (5-LOX). Other metabolites produced by LOXs are shown in yellow.

The hydrolysis at the sn-2 position of glycerophospholipids by PLA_2_s produces fatty acids and lysophospholipids. While lysophospholipids have potent biological catalytic activity and are the precursor for the platelet activating factor (PAF), one of the most biologically important fatty acids produced from this process is arachidonic acid (AA) (IUPAC name: (5Z,8Z,11Z,14Z)-5,8,11,14-eicosatetraenoic acid), which is further metabolised through enzymatic or non-enzymatic oxidation to eicosanoids that mediate diverse physiological responses. The two major classes of enzyme that directly metabolise AA are cyclooxygenase (COX) and lipoxygenase (LOX), whose products may be further metabolised by other downstream enzymes, that ultimately determine the type of eicosanoids produced. The metabolites produced from the COX pathway are collectively known as prostanoids and these are further categorised into prostaglandins and thromboxanes. The catalysis of AA by LOX enzymes produce hydroperoxyeicosatetraenoic acids (HPETEs) and some of these are further metabolised to leukotrienes. Several different types of prostanoids and leukotrienes exist and they mediate diverse biological effects. Figure 5 displays a simplified summary of the arachidonic acid cascade that lists some of the most important downstream inflammatory mediator products, but there are over a hundred eicosanoids, including the metabolites of cytochrome P450 (CYP) enzymes, that are important signalling mediators for the normal cell function [35]. hGIIA may hydrolyze the mitochondrial membrane and release AA, lyso-cardiolipin and mitochondrial DNA, which in turn can be recognized as damage-associated molecular patterns (DAMPs) and participate in pro-inflammatory pathways [36]. Although the catalytic activity of PLA_2_s initiates the eicosanoid cascade by liberating AA, the final biological outcome from this is heavily dependent on the types of downstream enzymes in the system.

The COX enzymes are also known as prostaglandin-endoperoxide synthases and their effects on pain, inflammation and control of body temperature are well established. Numerous pharmaceutical agents, that are designated as a class called non-steroidal anti-inflammatory drugs (NSAIDs) that inhibit these enzymes, have been introduced and widely used in clinic since the 19th century. These still remain as important pharmacological targets of pain and inflammation today. There are two subtypes of COX enzymes, COX-1 and COX-2, that are well characterised in human biology in terms of their structure and function. COX-1 is responsible for the production of thromboxane A_2_ (TXA_2_) in platelets, since platelets do not produce COX-2 [37], and irreversible inhibition with aspirin, which is selective towards COX-1 at a low dose, produces antiplatelet effects. COX-1 is constitutive, whereas COX-2 is inducible and regulated by cytokines, which previously made it an attractive target for inhibition.

Several prostanoids are known to be cytoprotective in the gastrointestinal tract. The mechanism of the cytoprotective effect of prostanoids is not monocausal, but rather is a combination of effects. They reduce the secretion of gastric acid by parietal cells, have vasodilation effects in the gastric mucosa, which increases the blood flow, and stimulate viscous mucus secretions in the stomach that act as a physical barrier to the gastric acid and help the formation of a layer of bicarbonate on the epithelium [37]. Classical COX inhibitors that non-selectively block both COX subtypes inhibit the beneficial housekeeping function on the gastrointestinal tract and may cause adverse effects such as dyspepsia and gastrointestinal bleeding and ulceration.

The identification of COX-2 [38] and development of its selective inhibitors, such as rofecoxib [39] and celecoxib [40], led to effective treatment of pain and inflammation in the clinical setting with reduced risk of developing gastrointestinal adverse effects. The effectiveness of these agents as anti-inflammatories can be attributed to their ability to reduce the production of prostaglandin E_2_ and I_2_ (PGE_2_ and PGI_2_, respectively). In rat models, deactivation of PGE_2_ with the monoclonal antibody 2B5 reduced both inflammation and pain [41]. The responses to inflammation and pain were significantly reduced in PGI_2_ receptor knockout mice, which demonstrate that PGI_2_ is an important inflammatory mediator [42]. However, selective COX-2 inhibitors increase the risk of adverse cardiovascular events when pre-existing conditions exist. Prostaglandin I_2_ (PGI_2_), more commonly known as prostacyclin, is a major prostanoid produced by endothelial cells that inhibits the contraction of the blood vessels [43]. Although several prostanoids interact in the homoeostasis of maintenance of blood pressure, the production of PGI_2_ impaired by NSAIDs may then induce or worsen hypertension. Moreover, selective COX-2 inhibitors can also have serious cardiac adverse effects due to their ability to induce thrombosis. The homeostasis of thrombosis is maintained by the balance of TXA_2_, which is prothrombotic and PGI_2_, which is antithrombotic. The disruption of this balance by the selective inhibition of COX-2 decreases the amount of PGI_2_ in the system and leads to thrombotic events. The Vioxx Gastrointestinal Outcomes Research (VIGOR) study [44] has indeed shown that over a 12 month period, the group taking rofecoxib had a 4-fold increase in the risk of developing myocardial infarction in comparison to the group taking naproxen, and consequently this particular inhibitor was withdrawn from the market. A meta-analysis study concluded that the selective COX-2 inhibitors, as a class, increased the risk of major vascular event by 37% compared to placebo [45]. However, in a more recent study, celecoxib was found to be non-inferior to ibuprofen, a non-selective NSAID in the overall cardiovascular safety [46]. There is now a general consensus that all NSAIDs other than aspirin, regardless of selectivity, may pose a serious risk of cardiovascular events, though the degree of risk may vary significantly between the NSAID drugs [47]. Due to the adverse effects and contraindications, there is a clear need for agents that target other proteins in the AA cascade than the COX enzymes to treat the diseases in which this pathway is implicated.

Of several subtypes of LOX enzymes found in humans, the 5-LOX subtype is perhaps the most implicated in inflammatory disorders. These enzymes oxidise AA and other fatty acids to produce a group of metabolites called leukotrienes, which are important lipid signalling mediators in inflammation, particularly in the respiratory systems. With the exception of leukotriene B4, leukotrienes exert their biological action by agonising the cysteinyl leukotriene receptors, and antagonists of these receptors are clinically used for the treatment of obstructive airway diseases, particularly asthma. Interestingly, there is a study showing that the production of hGIIA is at least partially regulated by 12-LOX and 15-LOX enzymes in conjunction with the group IVA cytosolic PLA_2_ (cPLA_2_-α) in rat fibroblastic 3Y1 cells [48]. This suggests a potential role of LOX enzymes in the cross-talk signalling with some PLA_2_ enzymes.

While there is a consensus that cPLA_2_-α is the main direct contributor to the AA production in the cytosol in mammalian cells [49,50,51], the role of hGIIA in provision of AA is less clearly defined. From a comparative study of several exogenously added sPLA_2_s to mammalian cells, it was seen that a strong correlation exists between the ability of the sPLA_2_s to hydrolyse PC-rich vesicles and the ability of these enzymes to release AA into the extracellular space [29]. PC is the major component of the outer leaflet of the phospholipid bilayer in mammalian cells. As hGIIA is secreted into the extracellular space, and thus has access only to the very outer layer of the cell membrane which consists primarily of PC, for which hGIIA has a low affinity. Hence, it is questionable whether hGIIA may efficiently hydrolyse the substrate to provide AA to make a significant contribution in the eicosanoid pathway.

Even so, several studies support that hGIIA has an important indirect role in releasing AA, and therefore is highly implicated in the inflammatory diseases induced by the eicosanoid pathway. For example, the exogenous addition of the GIIA significantly increased PGE_2_ generation in the presence of interleukin 1*β* (IL-1β) and tumour necrosis factor alpha (TNF), and also induced expression of both cPLA_2_-α and COX-2 in the mouse preosteoblast MC3T3-E1 cell line [52]. Similarly, the exogenous addition of hGIIA to human rheumatoid synoviocytes induced up-regulation of the cytokine-induced production of PGE_2_ [53]. Further work in this model system established that exogenous hGIIA mediated AA release into culture medium in the absence of cytokine stimulation, but that this release was likely derived from extracellular vesicles released by the synoviocytes rather than the cells [54]. In another study, overexpression of mammalian GIIAs in human embryonic kidney (HEK) 293 and Chinese hamster ovary (CHO) K1 cells mediated delayed AA release while overexpression in larger amounts mediated immediate AA release [55]. As hGIIA is unable to efficiently release AA inside or outside the cell, it may stimulate AA release through a catalytically independent mechanism.

### 4.2. Physiological Role of hGIIA

The expression pattern of hGIIA under normal physiological conditions is largely limited to secretory glands such as tear ducts, salivary glands, Paneth cells in the gut, the prostate and seminal vesicles, the lactating breast and gestational tissues. In addition, hGIIA is stored in vesicles in mast cells, platelets and eosinophils and is released on activation. As a result, the dominant physiological role of hGIIA is considered to be in host defense.

Because hGIIA is secreted to the extracellular space and exerts selective catalysis towards anionic phospholipids, the enzyme can perform selective lysis of bacterial cell membranes, in which the external layer is composed of anionic phospholipids. This selective catalysis provides protection from the attack of the human cell membrane, in which the external layer is composed of zwitterionic phospholipids. Multiple lines of evidence suggest hGIIA plays an important role in innate immunity by providing protection against bacterial infections. Such bactericidal activity was first reported against the gram-negative Escherichia coli, in the presence of the 54 kDa bactericidal permeability-increasing protein (BPI), which disturbs the lipopolysaccharide coat on the outer membrane, to allow the GIIA enzyme to penetrate to the underlying anionic phospholipid membrane before hydrolysis [56]. In the case of gram-positive bacteria, hGIIA is able to penetrate and access the anionic phospholipid molecules directly. Potent bacteriolytic activity of hGIIA has been demonstrated against multiple species, including Staphylococcus aureus and Listeria monocytogenes, and activity was significantly reduced in the presence of the anti-GIIA antibody [57]. Although GIIAs are not the only sPLA_2_ that have bactericidal activity, they display the highest potency against Listeria monocytogenes and Staphylococcus aureus, with the rank order as follows: IIA > X > V > XII > IIE > IB = GIIF [30]. This implies that bacteriolysis is one of the prominent roles of hGIIA, requiring only 0.5 μg/mL concentration to kill over 99% of these two species over 20 min exposure in vitro. This explains the reason for the high level of expression of the enzyme in specific locations at normal physiology, such as in human tears where it is found in concentrations exceeding 30 μg/mL [58]. It is observed to potently and selectively cause lysis of the gram-positive bacteria without affecting the corneal epithelial cells, although it was ineffective against gram-negative bacteria in the absence of bacterial permeability inducing protein (BPIP) [59]. Reviews of hGIIA as it relates to host defense have recently been provided [36,60]. The role of hGIIA with respect to innate immunity and resistance mechanisms are potentially complex and do not necessarily relate only to the bacterial membrane composition, and the role of GIIA in the microbiome could be a very important contribution to beneficial bacterial colonization. The effect on the microbiome includes the eradication of competitor Gram-negative bacteria at the mucosal surface [60].

### 4.3. Pathological Role of hGIIA

#### 4.3.1. Inflammation

Marked induction of the enzyme is observed in multiple pathological conditions with an inflammatory component. The enzyme gets released in response to proinflammatory cytokines such as interleukin-1β and tumour necrosis factor (TNF) [61], and is found in numerous inflammatory fluids and plasma in the pathological situations.

As the enzyme was discovered and characterised from the synovial fluids of the arthritic patients [8,9,10,11], the association of hGIIA with the pathophysiology of rheumatoid arthritis (RA) is foundational in the literature. In arthritis, hGIIA is markedly up-regulated and the concentration found in the synovial fluids can be up to several micrograms per millilitre [62]. hGIIA may serve as an important biomarker of RA, as clinical studies have concluded that there is a proportional relationship between the serum hGIIA concentration and disease severity [63,64,65]. Interestingly, other subtypes of sPLA_2_s are also expressed in the synovial fluids of RA patients, but it is the hGIIA subtype that is mainly responsible for the proinflammatory effects, whereas the group V subtype has been found to have opposing effects [66].

In several acute inflammatory conditions such as pancreatitis [67,68], peritonitis [69] and sepsis [70,71,72], elevated serum hGIIA level were observed. The overall level of hGIIA in individuals without inflammatory disease is low, but in response to acute inflammatory disease, the serum level may quickly rise up to 1000-fold within the first few days of the onset of the active disease. In the case of sepsis, the hGIIA serum level correlates with survival rate as, in a study, the group of patients who survived the disease had a significantly lower serum hGIIA level than the group who did not [71,72]. A recent review highlights the potential for the use of hGIIA as biomarker for sepsis in adults [73]. The protein levels are elevated in some inflammatory conditions in the respiratory tract. Both hGIIA and AA levels were found to be increased in bronchoalveolar lavage (BAL) fluid in patients with asthma after antigen inhalation challenge [74]. Functionally, hGIIA has the ability to degrade the phospholipid lung surfactant [75]. The hGIIA level in BAL fluid was elevated in patients with acute respiratory distress syndrome (ARDS) [75,76,77] and the level was correlated with the severity of the disease.

#### 4.3.2. Atherosclerosis

Atherosclerosis and related cardiovascular diseases are also conditions linked with the induction of hGIIA. Lipid biomarkers in blood have long been known for their role in the development of atherosclerotic plaque, but the more recent evidence suggests a proatherogenic role for various sPLA_2_ enzymes including hGIIA. Early studies using immunohistochemistry established that hGIIA was expressed in atherosclerotic plaque [78]. Although hGIIA has poor catalytic activity towards the PC of low-density lipoprotein (LDL), modification of PC by reactive oxygen species produce oxidised LDL particles which are then susceptible to hydrolysis by hGIIA [79]. Depletion of phospholipids in LDL leads to the formation of small dense LDL particles, which are proatherogenic [80]. The hydrolytic reaction produces high local concentrations of free fatty acids and lysophospholipids in the plaque, which mediate various inflammatory reactions. Along with other inflammatory markers, namely interleukin 6 (IL-6) and C-reactive protein (CRP), the level of hGIIA sharply increases during the first four days after acute coronary syndrome (ACS) [81]. Potentiation of the effects of oxidized LDL has been observed to occur through hGIIA in a pro-atherogenic manner by the activation of smooth muscle cells and secreting monocyte chemoattractant protein-1 [82]. Plasma levels of hGIIA are closely related to the severity of the outcome in ACS patients, as it has been seen that the probability of being subject to another coronary event after unstable angina and percutaneous coronary intervention was significantly increased in the cohorts with an elevated hGIIA level [83,84]. These biomolecular models and clinical results indicate that hGIIA is not only a suitable prognostic marker in atherosclerosis but also a potential therapeutic target. However, efforts to evaluate the therapeutic benefit in targeting hGIIA enzyme activity with a potent but non-selective sPLA_2_ inhibitor in Phase III clinical trials in patients with acute coronary syndrome have been unsuccessful due to a lack of efficacy and increased risk of serious adverse cardiac events [85], indicating the need for selective hGIIA inhibitors and a greater understanding of the functional role of sPLA_2_ enzymes in the development of coronary artery disease.

#### 4.3.3. Cancer

Elevation of the serum concentration of hGIIA in at least a proportion of patients has been observed in multiple types of advanced cancers, including lung, bile duct, prostate, stomach, liver, breast, oesophagus, colon and pancreas [86,87]. However, their biological role may be highly dependent on the type of the cancer cell and the location of tumour. For example, in gastric adenocarcinoma, the expression of hGIIA was found to be associated with prolonged patient survival and less frequent metastasis [88,89], suggesting that there is a potential anti-tumourigenic effect of the protein. On the other hand, a pro-tumourigenic role for hGIIA was suggested in prostate cancer, as the level of expression of hGIIA was associated with a higher grade of prostatic intraepithelial neoplasia [90]. Table 2, adapted from Brglez et al. 2014 [91], outlines some of the current known cancers in which hGIIA is involved and the hGIIA association with patient survival.

Multiple studies indicate that hGIIA is actively secreted by many of prostate cancer cell lines and its concentration in the tissue specimen and seminal plasma is elevated in prostate cancer and increases with the advancing stage of the cancer [90,98,99], although some cell lines, such as DU145, are hGIIA-negative [86]. It has been suggested that hGIIA could serve as an alternative biomarker to determine the prognosis of prostate cancer, as the serum concentration of hGIIA is closely correlated with the Gleason score [87], which is a histology biopsy-based method that is currently utilised in the clinic, though other observations suggest this is not the case, and, additionally, observations of elevated levels of hGIIA in benign prostate hyperplasia have been made [100]. Elevated serum hGIIA has also been found in patients with other malignancies such as head, neck, hepatic, pancreatic, myeloma and non-Hodgkin’s lymphoma [91,96].

Aside from use as a biomarker, hGIIA could also be an important therapeutic target in prostate cancer. Malignant prostate cancer cells have altered phospholipid remodelling and arachidonic acid metabolism, as the overall PLA_2_ activity was found to be 2-fold higher in malignant cells than benign cells and was accompanied by a 10-fold increase in PGE_2_ synthesis rate [101,102]. As eicosanoids promote tumour growth and metastasis [103], it is logical to relate the increased eicosanoid production with the increased expression of hGIIA. Indeed, the hGIIA mRNA expression level can be 22 times higher in prostate cancer cells than normal cells [104]. EGFR/HER2-PI3K-Akt and NF-κB pathways are involved in regulation of hGIIA gene expression as inhibitors of these pathways, such as Lapatinib, LY294002 and Bortezonib, downregulated the hGIIA expression at the transcriptional level [87]. hGIIA induces prostate cancer cell proliferation, as it has been observed that the exogenous addition of hGIIA dose-dependently stimulated the growth of LNCaP cells, even at doses as low as 1 nM [104]. The inhibitors of hGIIA were able to supress the growth of hGIIA-positive LNCaP and PC-3 cell lines but not the hGIIA-negative DU145 cell line [104]. This suggests that hGIIA is an important mediator of cancer cell proliferation, and modulation of its biological activity can suppress growth of the cancer cells. Interestingly, the hGIIA expression level in the androgen-independent LNCaP cell line is higher than the normal androgen-dependent LNCaP cell lines [87]. PLA_2_ activity is also the rate-limiting step in eicosanoid production under physiological conditions. Therefore, hGIIA should be a useful therapeutic target, especially for the androgen-independent and hGIIA-positive prostate cancers that are non-responsive to androgen ablation therapy.

## 5. Catalysis-Dependent and -Independent Roles of hGIIA

Snake venom sPLA_2_s, which have a high amino acid sequence identity with an hGIIA ranging from 30 to 60%, exert their biological actions by two discrete mechanisms, which are catalysis and protein–protein interaction [105,106]. These proteins produce more widely diverse physiological effects than the mammalian sPLA_2_ types, including neurotoxicity, myotoxicity, cardiotoxicity, platelet aggregation and anticoagulant effects [105,106]. It is suggested that while the catalytic activity is mainly responsible for aiding the digestion of lipids in prey, other toxicological effects have been attributed to the interaction with different protein targets via a separate binding site distinct from the substrate binding site [106,107]. Considering the structural similarities between snake venom sPLA_2_s and hGIIA, it is not a surprise that hGIIA could also exert at least some of its biological actions through mechanisms independent of catalysis.

It was possible to dissect the effects of the catalysis-independent mechanism of hGIIA by using catalytically inactive hGIIA mutants. Such mutants were created by making a single point mutation on the active site histidine. Neutralisation by the His48Asn mutation altered the protein such that it exhibited less than 0.5% of the catalytic activity of the wild type [108]. The charge-neutralised mutant of hGIIA, His48Gln, was reported to have 1–4% of catalytic activity compared to the wild type protein [54]. The crystal structure (PDB ID 1N28) confirmed that the His48Gln mutant had maintained the important structural features and the conformational integrity as they were virtually superimposable with the wild type protein (PDB ID 1N29) [108]. Therefore, any biological effects measured from the exogenous addition of this mutant can be anticipated to be dominated by a catalysis-independent mechanism. Another example is the Gly30Ser mutant, which is a mutation of the Gly30 of the calcium binding loop, and accordingly no longer capable of binding the catalytic calcium ion, and therefore incapable of proceeding with catalysis [109].

Several studies provide evidence that hGIIA or other GIIAs may contribute to the eicosanoid pathway by a means other than by directly providing AA through catalysis. As seen with wild-type hGIIA, exogenous addition of the hGIIA His48Gln mutant to fibroblast-like synoviocytes (FLS) obtained from patients with rheumatoid arthritis did not have a significantly altered ability to promote PGE_2_ production and COX-2 expression in the presence of TNF without activation of NF- B or p38 mitogen activated protein kinase (MAPK) signaling but with activation of the ERK MAPK pathway [54]. In another study using rat mesangial cells, both the wild type and His48Gln mutant enzymes were able to facilitate TNF-induced GIIA expression at the mRNA and protein levels, suggesting that such self-induction via an autocrine feedback loop is independent of catalytic mechanism [110]. In a study that used the rat Gly30Ser mutant, the protein was able to up-regulate COX-2 expression in rat serosal connective tissue mast cells under cytokine stimulation, but it was not able to induce AA release [109]. Similar results were observed in a study using CHO-K1 cells that were transfected with the wild type or the His48Gln mutant of hGIIA, as it was seen that the catalytically inactive variant hGIIA was not able to induce AA release in these cells [111]. Therefore, there is a consensus that hGIIA can induce COX-2 expression under the conditions of cytokine stimulation by a catalytically independent mechanism. It is also notable that the His48Gln mutant induced cell proliferation and ERK1/2 activation in monocytic cells, suggesting that proliferative signalling is induced independent of the catalysis [112].

There is also evidence that the ability of hGIIA to induce eicosanoid production or COX-2 expression may involve cPLA_2_-α and certain kinases, but data on the mechanism are conflicting. AA release was enhanced with expression of GIIA in murine mesangial cells (MC) but not in the cPLA_2_-α knockout MC, showing that GIIA has a regulatory role in the activation of cPLA_2_-α, while cPLA_2_-α is directly involved in the release of AA [113]. Conversely, it was seen that cPLA_2_-α induces the expression of the GIIA and COX-2, which in turn increased production of PGE_2_ in rat 3Y1 fibroblasts [114]. It can be seen that there is a cross-talk mechanism between the two proteins, which has an amplifying effect on each other. Such cross-talk mechanisms may involve an autocrine cycle where GIIA induces the expression of itself, providing the amplification effect. cPLA_2_-α and peroxisome proliferator-activated receptor α (PPARα) were found to be involved in this autocrine cycle to facilitate TNF-induced GIIA expression in rat mesangial cells [110]. In another study, it was found that PGE_2_ production was amplified by a PGE_2_ autocrine mechanism and the cPLA_2_-α and exogenous addition of the GIIA further enhanced the PGE_2_ production in mouse osteoblast cells [52]. Treatment with hGIIA in human lung microvascular endothelial cells (LMVEC) led to activation of the MAPK/ERK pathway, which was suggested as the potential mechanism of the activation of cPLA_2_-α by hGIIA [115]. Stable expression of hGIIA in CHO-K1 cells led to the interleukin 1β-dependent release of AA but this was completely dependent on cPLA_2_-α [111]. Interestingly, exogenous addition of hGIIA had lesser effects on the release of AA compared to the stable expression, suggesting that hGIIA needs to be located in the intracellular space to promote AA release [111]. It can be seen that there is a consensus on the existence of the cross-talk between the GIIA and cPLA_2_-α, but the exact effect and mechanism may be highly specific to the cell type and the species from which the cells were derived. Although cPLA_2_-α is the enzyme ultimately responsible for the release of AA, hGIIA is considered a more attractive target to minimise eicosanoid production due to its upregulation under pathological conditions, as hGIIA mRNA was found to be 22 times higher in prostate cancer than normal prostate [103]).

## 6. Binding Partners of hGIIA

It is convincingly established that hGIIA does not exert its biological actions through its catalytic mechanism alone, and, very importantly, involves signalling with multiple members of the inflammatory pathway. Such catalysis-independent mechanisms may be attributed to direct interaction or binding with another, yet-to-be-determined, molecule or molecules. In some instances where the catalytic activity is a prerequisite for the biological effects, it may also be an additional requirement for the protein to migrate into the intracellular space. Therefore, in such a case, hGIIA would need to interact with other molecules to achieve such transport. Together, these ideas led to a search for potential targets that may interact with hGIIA as part of a mechanism promoting the activation of various inflammatory kinases.

### 6.1. M-Type Phospholipase A_2_ Receptor

M-type phospholipase A_2_ receptors (PLA_2_Rs) were first identified in rabbit skeletal muscle tissue as a protein that has high affinity to two iodinated PLA_2_ monochains known as *Oxyuranus scutellatus* toxins 1 and 2, which are of snake venom origin [116]. This 180 kDa type I transmembrane glycoprotein is only composed of one subunit, with a large portion of the protein sitting extracellularly and a short tail of 40 amino acids in length on the C-terminal region found in the cytoplasm [116]. The cloned rabbit PLA2R was found to have 29% sequence identity to the human mannose receptor, while the interspecies sequence identity between bovine, rabbit, mouse, and human types is over 70% [117,118].

It has been suggested that the PLA_2_R has a major regulatory role in the control of sPLA_2_ concentration. Indeed, the cytoplasmic domain of PLA_2_R has an endocytosis motif Asn-Pro-Xxx-Tyr, which has been shown to facilitate the internalisation upon binding of porcine group IB PLA_2_ (pGIB), which then proceeds to degradation of the pGIB ligand [119,120]. PLA_2_R may also act as an endogenous inhibitor of sPLA_2_ when it is found in a soluble form in the circulation. Cleavage at the transmembrane tether of PLA_2_R releases the extracellular domain which retains identical binding properties as its parent membrane-bound protein [121]. There is also a report of two transcript variants of PLA2R in human kidney, where the encoding ratio between the full transmembrane protein and the alternatively spliced soluble protein was 1.6:1 according to quantitative polymerase chain reaction (qPCR) experiments [122]. In the case of group IB PLA_2_ enzymes, some of its biological activities that were independent of catalysis were strongly associated with binding with PLA_2_R, as both active and inactive forms of pGIB were able to activate p38 mitogen-activated protein kinase (MAPK) in neutrophils upon binding to PLA_2_R [123]. In another study, methyl indoxam, which inhibits binding of pGIB and its catalytically inactive mutant His48Gln to PLA_2_R, was able to suppress the production of TNF and IL-6, which were inducible by the both active and inactive pGIB [124].

However, it is unreasonable to directly generalise the results of studies using group IB PLA_2_ to be directly applicable for hGIIA. The binding specificity of mouse PLA_2_Rs is limited to only a subset of sPLA_2_ enzyme subtypes. Such binding also exhibits specificity for species, as human PLA_2_R did not recognise hGIIA as its ligand, while the rabbit counterpart demonstrated specific binding [122,125]. This shows the overall biological function of PLA_2_R may vary significantly between species. Considering that the physical binding of hGIIA and PLA_2_R does not occur, it is unlikely that hGIIA would exert its biological action in association with PLA_2_R.

### 6.2. Heparan Sulfate Proteoglycans

Heparan sulfate proteoglycans (HSPGs) are glycoproteins found on the cell surface and function as receptors of ligands to promote multiple cellular actions, such as endocytosis, cell adhesion and signalling [126]. Multiple lines of evidence correlate with the notion that the binding of hGIIA to HSPGs is a critical step in mediation of the eicosanoid pathway. In rat BRL-3A cells, the ability of GIIA to generate PGE_2_ was reduced by the extracellular addition of heparin or pre-treatment of the cells with heparin-sulfate lyase [127]. As exogenously added heparin removes the GIIA bound to HSPG, as it has high affinity for cationic proteins and the heparin-sulfate lyase degrades the HSPG on the cell surface, it is evident that HSPG binding of the enzyme is an important step in the production of prostanoids. This observation was also seen in HEK293 cells, as treatment with heparin, heparin-sulfate lyase or phospholipase C resulted in the dissociation of membrane-bound hGIIA into the cell media, demonstrating that hGIIA is indeed bound to the cell surface via HSPG [128]. The overexpression of each of hGIIA and glypican-1, a glycosylphosphatidylinositol (GPI)-anchored HSPG in HEK293 cells, increased AA release, PGE_2_ production and COX-2 expression under interleukin-1 stimulation and this induction of COX-2 occurred in a synergistic manner when the two proteins were coexpressed [128]. Similarly, the binding of hGIIA to HSPG was associated with the release of AA from human apoptotic T cells [129].

It is clear that hGIIA binds to cellular HSPG and associates with the cell surface to trigger the production of prostanoids, but the process is highly dependent on the activity of cPLA_2_-α, rather than the activity of hGIIA itself alone [130]. The mechanism of regulation of prostanoid production by HSPG was found to be associated with the internalisation of hGIIA via potocytotic vesicle transport into the perinuclear area. Immunofluorescence microscopy studies suggested that hGIIA was localised in the caveolae, where GPI-anchored proteins, COX-1, COX-2 and cPLA_2_-α are also in the vicinity [128]. It was proposed that this internalisation and localisation facilitated by HSPG is a prerequisite for hGIIA to activate cPLA2-α via phosphorylation in the cross-talk signalling that results in AA release [55,131]. Notably, the release of AA was reversed by the addition of heparin, but not by the inhibition of catalysis by *p*-bromophenacyl bromide (BPB) in the human astrocytoma cell line 1321N1 [131]. BPB irreversibly binds to the histidine of the catalytic dyad and inactivates the catalytic ability without affecting the overall conformation or the chemical and physical characteristics of the protein surface. Considering that heparin abrogates hGIIA interaction with HSPG, it can be suggested that hGIIA is closely associated with HSPG in mediating cPLA_2_-α activation and such action is independent of its catalytic activity.

In THP-1 cells, the binding of hGIIA to HSPG induced macropinocytosis by forming a large vesicle composed of anionic phospholipids, which indicates that hGIIA may have a physiological function in removal of extracellular cell debris and microparticles that may be produced under inflammatory conditions [132]. It was observed that fluorescently labelled hGIIA was internalised to the cell nucleus through this HSPG-dependent endocytosis mechanism and this process did not require the catalytic activity of the enzyme [132].

Unlike binding to phospholipid aggregates where the interfacial binding surface plays a key functional role, the binding of hGIIA to the anionic HSPG is driven by the overall basic charge of the protein. This is confirmed by an in vitro study that tested 26 mutants of hGIIA that had charge reversal on different basic amino acids, as there were no specific localised sites of basic amino acids, but instead diffused HSPB binding sites that partially overlap with the interfacial binding surface [133]. Interestingly, the catalytic activity of the charge reversal mutants was dependent on their affinity towards phospholipid vesicles but not heparan sulfate [133]. Therefore, the binding of hGIIA to HSPG seems to be dependent on electrostatic interactions originating from the overall charge of the enzyme rather than involvement of specific sites, and independent of catalytic activity.

Overall, it can be seen hGIIA function mediated by binding to the HSPG, internalisation via endocytosis and activation of cPLA_2_-α is independent of catalytic ability.

### 6.3. Integrins

Integrins are cell adhesion receptors that recognise extracellular matrix proteins and cell surface ligands. They are heterodimers composed of two subunits, namely α and β, and possess an extracellular headpiece domain to which a ligand can bind and cause a gross conformation change which, in turn, transduces signals through intracellular space [134].

In the search for a receptor of hGIIA on the cell membrane, it was demonstrated by Saegusa et al. that hGIIA binds to integrin ανβ3 and α4β1, using multiple assay techniques, including immunoassay, fluorescein labelling and surface plasmon resonance (SPR) [112]. Interestingly, proteoglycan-deficient CHO cells expressing transfected human β3 integrin bound to immobilised hGIIA compared to mock-transfected CHO cells, confirming that this specific binding is associated with integrin rather than HSPG [112]. The docking simulations between hGIIA and integrin ανβ3 in the same study identified that the integrin binding interface of hGIIA was independent of the catalytic site, and included the Arg81 and Arg108 amino acids. Mutation of these two residues via charge reversal abrogated the integrin interaction on SPR [112]. The wild type hGIIA was able to induce monocytic U937 cell proliferation via ERK1/2 activation while the charge reversal double Arg74Glu/Arg100Glu mutant did not [112]. Since this region of hGIIA has little functional relevance to its catalytic activity, it can be seen that one of the catalysis-independent roles of hGIIA is to transduce proliferative signals via the ERK1/2 pathway by firstly binding to integrins.

Interestingly, the hGIIA binding sites of integrins ανβ3, α4β1 and α5β1 are distinct from the classical RGD-binding site that recognises the RGD motif of the ligand [135]. The binding site of integrins that hGIIA binds was the same site that a cytokine protein named fractalkine binds to, which activated integrins via an allosteric mechanism [135,136]. Therefore, it was suggested that hGIIA binding activates integrin in an allosteric mechanism, which may possibly only involve local conformational changes in the headpiece domain [135]. The peptides derived from the hGIIA binding site of β1 and β3 subunits of integrin acted as inhibitors of the interaction between hGIIA and integrins α_ν_β_3_ and α_4_β_1_ [135]. Furthermore, inhibitors of the binding of hGIIA to integrin α_ν_β_3_ were developed from peptide libraries, and a tetrapeptide molecule linked with pyrazolylthiazole moiety was found to have IC_50_ of 20 μM according to their cell adhesion assay using immobilised hGIIA [137]. The docking simulation, as part of this study, predicted that this inhibitor interacts with Arg81 and Arg108 of hGIIA, rather than to the integrin [137].

However, the biological assays measuring the interaction between hGIIA and integrins required a concentration of more than 5 μg/mL of hGIIA before integrin could be activated [135]. The only biological tissue or fluid where the local concentration is known to be higher than 5 μg/mL is in human tears, where the concentration exceeds 30 μg/mL [59], or possibly in serum from patients with severe sepsis [70]. Although it may be reasonable to assume integrin activation by hGIIA may have important and unique biological role in tears, perhaps as an adjunct to its bactericidal role via catalysis, it is difficult to conclude the same necessity in other tissues where the local concentration of hGIIA falls well below 5 μg/mL. hGIIA may interact with integrins under pathological conditions as it can be upregulated to concentrations above 5 μg/mL.

### 6.4. Vimentin

Vimentin is a 53.7 kDa protein expressed mainly in the mesenchymal cells. This type III intermediate filament protein is composed of three domains where the highly conserved rod domain is flanked by the basic head and acidic tail domains [138,139]. The head and tail domains are highly flexible segments and do not participate in the formation of secondary structures, whereas the rod domain is composed of a series of highly conserved α-helices interrupted by linkers and participates in interactions with another vimentin molecule to form a coiled-coil dimer [138,139,140]. The coiled-coil dimer then forms a tetramer half-staggered in the anti-parallel orientation, and these tetramers laterally assemble to form a unit-length filament (UMF) composed of 32 monomers that elongate to form a mature filament [141,142]. The filament is highly stable in high ionic strength environments and disassembles only under low salt conditions [138].

The primary physiological function of vimentin is in the maintenance of the integrity of the cell in the cytoskeleton, interacting with microtubules and microfilaments. Vimentin knockout mice develop and reproduce normally without any apparent abnormalities, suggesting that it has no essential role in embryonic and post-natal development [143]. However, they were less resilient to physiological stress, as induced reduction of renal mass by ablation was lethal in the vimentin knockout mice while no lethality was observed in the control wild type group [144]. Vimentin knockout mice also had altered structural responses in arteries in shear stress to them, induced by change in blood flow [144]. Vimentin therefore may not be essential for reproduction and development at the early stage, but has an important survival role in withstanding the mechanical stress and recovery from injuries.

The intracellular vimentin network extends outward from the centre of the cell radially but also is highly dynamic in nature, as the filaments constantly remodel their shape [145]. Vimentin has high intracellular motility and may be transported along the microtubules bi-directionally, either towards the nucleus or periphery of the cell, promoted by dynein or kinesin, respectively [145]. Such a process may involve phosphorylation of vimentin on the Ser38 and Ser72, as this was observed to shift the equilibrium towards disassembly of the filament [146].

Despite being a cytoskeletal protein, vimentin accounts for 7.6% and 13.5% of total proteins on the surface of cardiomyocytes and vascular smooth muscle cells respectively, as measured by biotinylation [147]. It was suggested that integrin β3 has a role in recruiting vimentin to the cell surface [148], and activated macrophages secreted vimentin into the extracellular space [149]. The rod and a part of tail domains of vimentin were found to be exposed on the surface of apoptotic human T cells, which allows for the binding of extracellular hGIIA [150]. Contrary to this notion, there is a report that vimentin interacts with the membrane through its head domain in a study using erythrocyte membrane vesicles [151]. It is possible that the particular domain of vimentin exposed to the extracellular space may be specific to the cell type, but there is agreement that vimentin dynamically translocates to the cell surface and interacts with other extracellular molecules. Upon the interaction with another protein, vimentin may transduce an array of signals to the intracellular space. The vimentin signalling response is highly dependent on the interacting protein. Activation of the ERK1/2 MAPK pathway, cell migration and intracellular lipid transport are just a few examples [152].

Vimentin was suggested as a possible binding partner of hGIIA, as it was coimmunoprecipitated with a 57 kDa protein localised in the cytoskeleton of human apoptotic T cells that was identified as vimentin through peptide mass fingerprinting using matrix-assisted laser desorption/ionization time of flight mass spectrometry (MALDI-TOF MS) [150]. Far-Western blotting and enzyme-linked immunosorbent assay (ELISA) experiments using recombinant vimentin fragments of each domain revealed that hGIIA binds to the rod domain but not to the head or tail domains [150]. The binding was found to be independent of HSPG and heparin was not able to inhibit the interaction between the two proteins [150].

Immunofluorescence microscopy on FLS cells demonstrated that the exogenously added hGIIA was rapidly internalised and co-localised with vimentin [13]. The inactivation of catalytic function of hGIIA with BPB did not affect the co-localisation, suggesting that its interaction with vimentin is of an entirely catalysis-independent mechanism [13]. In the presence of cytokines, exogenously added hGIIA promoted PGE_2_ production but perturbation of the interaction between hGIIA and vimentin using inhibitors blocked the PGE_2_ production [13,54]. It is notable that the production of PGE_2_ following hGIIA stimulation appears dependent on cPLA_2_-α, based on pharmacological intervention with two structurally distinct inhibitors [54]. Interestingly, there are reports that vimentin, by utilising its head domain in the interaction, acts as a functional adaptor of cPLA_2_-α in the perinuclear region [153,154]. Therefore, it is tempting to suggest that vimentin may act as a functional adaptor that enables internalised hGIIA to activate cPLA_2_-α in the intracellular space to promote eicosanoid production. Furthermore, phosphorylated Erk1/2 MAPK (pERK) also binds to the second coil of vimentin [155], which protects the kinase from dephosphorylation as a result of calcium-dependent steric hinderance. It can be proposed the interaction between hGIIA and vimentin is the basis of at least some of the catalytic-independent mechanisms of hGIIA in eicosanoid production and could serve as an intervention target for the implicated inflammatory diseases. Such diseases could include cancer. In many epithelial cancers, such as prostate cancer, vimentin is found to be overexpressed. Vimentin serves as a marker for epithelial-mesenchymal transition (EMT) and its expression level is closely associated with the rate of tumour growth, invasiveness, and poor prognosis [152]. There is then the potential for pharmacological inhibition of the interaction between hGIIA and vimentin to improve the prognosis of cancer.

## 7. Inhibitors of hGIIA

**Figure 6:**
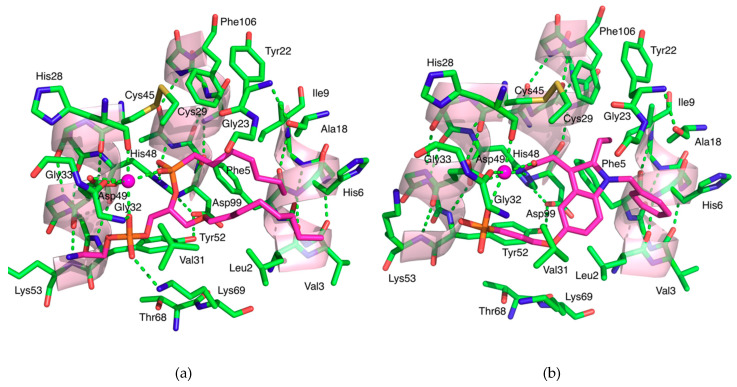
The active site and inhibitors bound from high-resolution crystal structures of hGIIA complexes. Amino acids are depicted if they are within 5 Å of any atom of either inhibitor or the catalytic Asp 99. In addition, contiguous elements of the representation of the main chain of 3 helices (transparent pink cartoon) are provided to aid in an orientation comparison with Figure 2. The carbons of the inhibitors and calcium are coloured mauve. The active site cavity opening is most obviously bounded by Leu2, His6, Ala18, Phe24 and Val31. The main chain of Cys29, Gly30 and Val31 have been removed from the foreground and, as a consequence, the coordination of the calcium by O(Gly 30) is not indicated in both structures or an N(Gly 30) interaction with a phosphonyl oxygen of the transition state analogue. (**a**) The transition state analogue (TSA) *L-1-O*-octyl-*2*-heptyl-phosphonyl-*sn*-glycero-*3*-phosphoethanolamine (PDB ID 1POE at 2.1 Å resolution [156]). The two hydrophobic chains of the TSA are relatively parallel, and this structure is the best understanding of the native substrate orientation at the active site channel. The end 3 carbons on the sn-1 chain were not determined, but nevertheless the sn-1 chain is in proximity to the Leu2, Gly30, Val31 and Tyr52, and the *sn-2* with Phe5, Ala18, and Gly23; (**b**) The LY311727 (PDB ID 3U8D at 1.8 Å resolution [13]). The two views have been chosen to be similar. For LY311727 the hydrophobic interactions are provided by Leu2, Phe5, His6, Leu20 and Gly30. Figure was created with PyMOL [14].

Early attempts at the development of effective inhibitors of sPLA_2_ started from the synthesis of the analogues of the phospholipid substrates. The first hGIIA crystal structure with a ligand to be deposited in the PDB (PDB ID 1POE, 2.1 Å resolution) including a transition state analogue (TSA) inhibitor called *L-1-O*-octyl-2-heptylphosphonyl-*sn*-glycero-3-phosphoethanolamine which was designed to be a mimic of the tetrahedral intermediate formed during the hydrolysis of *L-1,2*-dioctanoyl-*sn*-3-phosphatidylethanolamine, and has a phosphonate replacing the *sn*-2 ester [156]. The compound arose from studies surveying short chain substrates and inhibitors on a number of PLA_2_s [157], although not hGIIA. The TSA interacts closely with the primary calcium with the oxygens of two phosphates providing ligating atoms (see Figure 6a). A structure of the same TSA was reported prior to the description of the hGIIA complex with the GIIA from Chinese cobra (*Naja naja atra*) venom (PDB ID 1POB, 2.0 Å resolution) [158]. The positioning of the polar head groups is effectively the same as in the hGIIA with the two hydrophobic chains occupying the same space in both structures with some conformational differences, more especially identifiable at the start of the *sn-1* chain. As also found in the hGIIA-TSA complex, there is less confidence in the positions of the carbons in the hydrophobic chains towards their termini.

**Figure 7:**
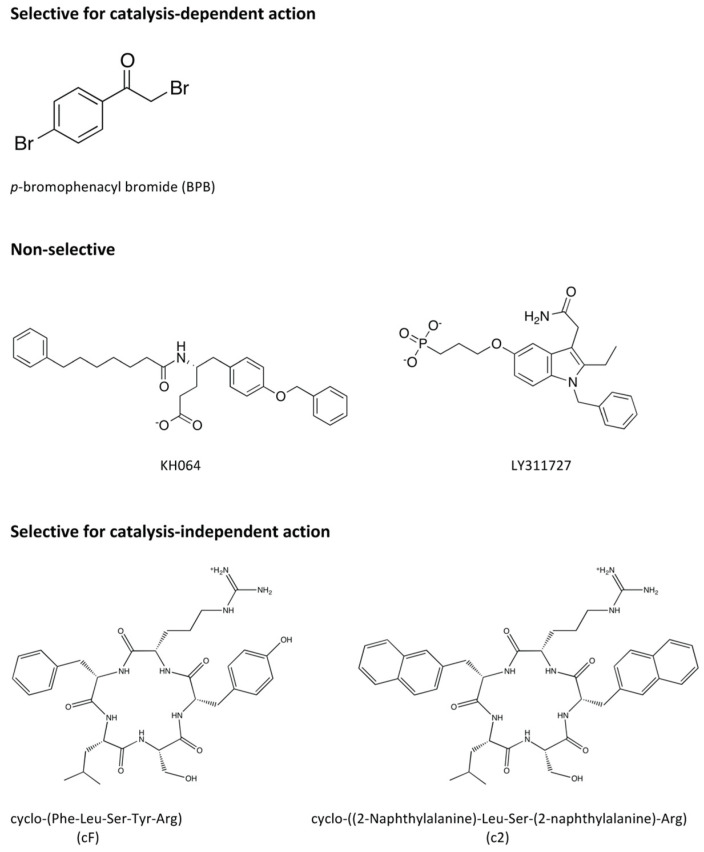
Several hGIIA inhibitors for which classification is known based on their functional mechanism of inhibition.

Schevitz and co-coworkers from Eli Lilly provided important structure-based relationships when they published three crystal structures with their indole-based designed inhibitors bound to hGIIA (accession codes 1DB4, 1DB5 and 1DCY; 2.2, 2.8 and 2.7 Å resolution) [159]. The subsequent development of sPLA_2_ inhibitors led to the discovery of an array of chemically diverse groups of compounds from several pharmaceutical companies and research groups. The classes of sPLA_2_ inhibitors have been reviewed previously [1,160]. The most conventional way of classifying these inhibitors would be based on their chemical structure, but Lee et al. proposed classification based on functional mechanism of inhibition [13]. From measuring the inhibitory effect on the catalytic activity with a spectrophotometric assay and the signalling activity through quantification of PGE_2_ end product on FLS cells, the tested inhibitors could be classified as one of the following: (1) selective for the catalysis-dependent mechanism, (2) non-selective or (3) selective for the catalysis-independent mechanism [13] (Figure 7). In addition, the displacement of the His6 sidechain was put forward as part of the mechanism of action for the inhibition of the catalysis-independent mechanism of hGIIA [13].

### 7.1. Inhibitors Selective for the Catalysis-Dependent Mechanism

*p*-Bromophenacyl bromide (BPB) (Figure 7) is an irreversible inhibitor of hGIIA that inactivates the enzyme through alkylation of the active site histidine, which is achieved through coincubation with highly purified enzyme in the experiments as the chemical reaction is not necessarily specific to hGIIA [161]. The crystal structure of the BPB-modified hGIIA (PDB ID 3U8I, 1.10 Å resolution) [13]) shows that the overall conformation and integrity is unchanged from the ligand-free hGIIA and the region occupied by the inhibitor does not extend to the surface. The treatment of hGIIA with BPB totally abrogates the catalytic ability of hGIIA, but the ability to transduce signals is unaffected [13].

### 7.2. Non-Selective Inhibitors

There were two inhibitors tested by Lee et al. that displayed inhibition towards both the catalysis and signal transduction mechanisms [13]. The first one was KH064 (5-(4-Benzyloxyphenyl)-4*S*-(7-phenylheptanoylamino)pentanoic acid) (Figure 7), an amide inhibitor that was developed as a D-tyrosine derivative [162]. There are two crystal structures of its complex with hGIIA at the PDB: 1J1A, 2.20 Å resolution [162] and 3U8H, 2.30 Å resolution [13]. The complexes have similarity but more recent evaluations suggested that, in the conformers of 1J1A, the atoms are less appropriately placed in comparison to 3U8H, which is also more particularly the case around the benzyl ether moiety [163].

The second compound is LY311727 (3-[3-(2-amino-2-oxoethyl)-1-benzyl-2-ethylindol-5-yl]oxypropylphosphonic acid), a structure for which it is in complex with hGIIA (PDB ID 3U8D, 1.80 Å resolution) is available [13] (Figure 2, Figure 6b and Figure 7). This compound was one of an array of indole-based inhibitors considered by Eli Lilly, but generally with the N-benzyl indole scaffold. Eli Lilly made depositions at the PDB of their indole compounds co-crystallised with hGIIA but without providing the LY311727 [159], but nonetheless giving important structural insights. The indole compounds are evidently related to indomethacin, the COX inhibitor widely used in the clinic, but the actual lead arose from a large-scale screen. These indole structures form interactions with hGIIA with similarity to the TSA inhibitors. The analogues did invoke a small but significant move in the indole location, and the substituents and the linker size at the 5 position of the indole were then considered to ultimately derive LY311727, with the phosphonate at the end of the linker also providing a calcium ligand (Figure 6b) [13,159]. The work continued to explore indole-3-acetamide-based inhibitors [164,165], but ultimately included a 3-glyoxamide series [166], and within this series 4-substitutions were found to generally be preferred over the 5-substitution seen in LY311727. Shionogi also contributed series of indolizine and indene versions of the Lilly indole compounds in this period [167,168]. The most promising candidate arising then came as LY315920, an indole, (also known as varespladib, A-001 or S-5920), with the 3-glyoxamide and 4-oxyacetic acid [166,169]. An orally active derivative and methyl prodrug LY333013 of LY315920 (varespladib methyl, A-002 or S-3013) was also put forward, in which the additional methyl is in a methoxy group terminating the 4-oxyacetic acid [170]. These were taken to clinical trials for the treatment of inflammatory diseases by Anthera Pharmaceuticals. Out of several trials, those for the treatment of atherosclerosis-related vascular diseases went further and continued to phase III, but the eventual outcome was negative as the treatment group taking varespladib methyl and atorvastatin combination therapy had no reduction in the development of recurrent cardiovascular events, but increased risk of developing myocardial infarction, in comparison to the placebo group who were given atorvastatin only [85]. An important feature of this drug is that in addition to inhibiting both the catalytic and non-catalytic functions of hGIIA, the inhibitor is not selective for hGIIA, but also potently inhibits GIIE, GIIV and GIIX [171]. The negative results may indicate that non-selective inhibition of hGIIA is not desirable for the treatment of inflammatory conditions, particularly in light of the data establishing that genetic ablation of sPLA-V in mice exacerbates inflammatory arthritis [66]. The trials highlighted that hGIIA may be a biomarker for inflammation but not the causal component of the progression of cardiovascular diseases [172]. However, little is known about the overall mechanism of the adverse effects, and it is desirable to discern the biological and clinical consequences of the selective inhibition of either catalysis alone or of the catalysis independent functions of hGIIA.

### 7.3. Inhibitors Selective for the Catalysis-Independent Mechanism

The pentapeptide FLSYK is a tryptic digest product of hGIIA comprising the 70th–74th residues of the parent protein [173], and at a relatively extended section of the polypeptide just before the -wing (Figure 1 and Figure 2). The peptide was found to be weakly inhibitory towards the parent protein hGIIA itself, and such native peptide inhibition also occurred with pentapeptides from the equivalent region for both the group IIA snake venom sPLA_2_s from *Crotalus durissus* and *Crotalus atrox* (pentapeptides WDIYR and TVSYT, respectively), but the inhibition was specific towards the parent enzyme from which the peptide was derived [173]. Modification of the FLSYK pentapeptide led to the conclusion that the cyclised peptide increased the potency by 5-fold [174]. This eventually led to the development of the c2 compound [cyclo-((2-Naphthylalanine)-Leu-Ser-(2-Naphthylalanine)-Arg)][cyclo-((2-Napthyl)alanyl-leucyl-seryl-(2-naphthyl)alaninyl-arginine] (Figure 7), which is a cyclised version with replacement of the aromatic amino acids phenylalanine and tyrosine with the highly hydrophobic 2-naphthylylalanine and the lysine replaced with an arginine, which resulted in further improvements in potency [174].

While these cyclic peptide inhibitors were potent inhibitors of the production of PGE_2_ in human rheumatoid fibroblast-like synoviocyte cells, the inhibition of hGIIA catalysis occurred at a concentration 1 × 10^6^ times in excess of the protein [13]. This cyclic peptide therefore selectively inhibited the catalysis-independent mechanism of hGIIA of PGE_2_ production without affecting catalysis of the protein. While it can be supposed that such inhibition is achieved through hGIIA interacting with a partner protein, such as vimentin, to prevent further signal transduction, there are no crystal structural studies currently reported that support how such inhibition could be achieved. Several group IIA snake venom sPLA_2_ co-crystallised with various peptide ligands are available in the PDB, but none is available for the human variant. There have been some previous attempts in the co-crystallisation of hGIIA with the cyclic peptides, but the electron density of the ligands could not be satisfactorily described [175].

## 8. Recent Developments for hGIIA

The classification of hGIIA and snake venom PLA_2_ as type IIA PLA_2_s made studies that cross over between the two proteins appealing, and the structure of the *Daboia russelli pulchella* snake venom PLA_2_ (svGIIA) with FLSYK bound was reported (PDB ID 1JQ9, 1.80 Å resolution) [176]). The FLSYK appears in a plausible pose in the groove over the entrance to the active site. The work would appear motivated by the reports of the FLSYK inhibition of hGIIA, though some inhibition data for the svGIIA were also provided [176]. As alluded to earlier, the X-ray crystallographic determinations of FLSYK co-crystallised with hGIIA have been difficult to achieve and would assist in the design of improved inhibitors, and also in comparisons with snake versions of PLA_2_. There continue to be efforts exploring the application of inhibitors to the snake venom PLA_2_, such as the recent work on the potential of the indole-based inhibitors in snake bite treatments [177], for instance. Another pentapeptide, LAIYS, also reported to be an inhibitor of the *Daboia russelli pulchella* GIIA and for which a structure became available (PDB ID 1JQ8, 2.00 Å resolution), but for which there is no publication and only a minimal and basic description of any design logic in the reports on the derivation of this pentapeptide. A search reveals that LAIYS is at the “70–74 location” of a chain of the PLA_2_ from the snake *Vipera ammodytes meridionalis*. The endogenous LAIYS peptide also occupies the similar location to the endogenous FLSYK in the superimposed protein chain structures. Unlike the work coming from the T.P. Singh laboratory on *Daboia russelli pulchella* GIIA, a very comprehensive screen was performed by Scott and co-researchers, which generally showed great sensitivity to single amino acid changes at each location in the FLSYK in their inhibition assays of hGIIA [174]. More recently, the veracity of the structure determination with bound LAIYS has been questioned, with the result that the peptide was considered not to exist in the structure [178], and a refinement with no peptide was the ensuing deposition to the protein databank (PDB ID 5VET; 2.00 Å resolution). A similarity in the FLSYK and LAIYS complex structures is that the peptides are placed bound to only one of the two protein molecules of the enzyme in the asymmetric unit. An altered conformation of the Trp 31 sidechain in the second molecule relative to the first is reported to be responsible for the FLSYK not binding in the second site, and yet a simple rotation of Trp31 away from the position to accommodate the FLSYK where it would otherwise be bound would seem very plausible [176].

Molecular dynamics (MD) calculations have the potential to indicate the misplacement of ligand atoms in crystal structures, as the criteria for ligand atom placement are often not as stringent as those for the protein atoms. MD simulation studies undertaken [163] firstly consolidated evidence that the behaviour of hGIIA is different in the solution environment relative to the crystal environment, in that it predicted that hGIIA would be stable as a monomer in physiological environments, implying that hGIIA dimers may not be relevant to the protein’s biological actions. Secondly it was seen that the structural differences between hGIIA and the GIIA of the snake *Daboia russelli pulchella* would not support analogous binding of a pentapeptide in the case of FLSYK. Importantly there is a sidechain absent from the region of the entrance of the active site pocket for the svGIIA, with a Gly at this position (Gly6), and it is the much more bulky and functionalised His6 is at this position in hGIIA. Ultimately it was concluded that the binding of the FLSYK molecule reported in the svGIIA in the crystal structure [176] does not have any value for predicting its binding conformation in hGIIA [163].

## 9. Recent Developments for Other Secreted PLA_2_s

As LY315920 (varespladib) strongly inhibits hGIIA, hIIE, hGV and hGX, finding selective inhibitors is one of the imperatives of the field, either for their potential as a therapeutic, or to assist in teasing out the biological mechanisms at play with these enzymes. The crystal structure of hGIIE, the sequence of which is most similar to hGIIA, became available in 2017, and along with it so too did structures of several indole-based inhibitor complexes, including LY311727 (PDB ID 5WZW, 1.95 Å resolution) [171]. The structure of hGIIE enabled comparisons with hGIIA and hGX. The overall structures of all these secreted PLA_2_s are similar, and the loss of charges from the C-terminal region and N-terminal residues in the hGIIA are easily structurally accommodated, as has been observed in these small PLA_2_ structures more generally. In addition to the neutrality of the C-terminal residues of hGIIE there is another patch adjacent to, but behind the i-face, which is also neutral relative to other PLA_2_s, and includes the Trp which is substantially conserved in human PLA_2_s but is Arg43 in hGIIA. The LY311727 structures of the complexes with hGIIA and hGIIE superpose well and there are no significant differences in the manner in which it binds. Although the overall changes in amino acids to neutrality may be considered consistent with the change in enzymatic substrate profile away from PG for hGIIE, this is consistent with the altered binding capacity which is also required for accommodating differing partner proteins in a mechanism independent of enzymatic activity. It is concluded that the second calcium binding site in hGIIE is unstable, or at least unstable relative to hGIIA, and the possibility of the second calcium as a backup store for the main calcium is put forward, largely on the basis of the observed varying calcium occupancies in the series of structures [171]. The second calcium binding site is, however, different to that of hGIIA in that the sidechain carboxylate of Asp24 (of hGIIE) is seen to have the ability to coordinate the calcium, whereas it is the mainchain carbonyl of the Phe24 in hGIIA at the same position in the sequence that is the ligand for the calcium ligation.

A wider entrance to the active site occurs in hGIIE due to a slightly altered positioning of Lys69 in hGIIA as well as the loop on which it sits. Similarly, relating to this entrance, the observation has been made for hGIIA that His6 swings out of this entrance to the substrate binding pocket, and additionally forms a hydrogen bond with Glu17 in the structure when LY311727 is bound [13]. In consideration of the requirements of inhibitors, one needs to be mindful that there is a small or no sidechain at this location in the case of hGIIE, at which there is a Gly, and for hGX, at which there is Ala, a location of interest and raised in this review, and as well there is the potential for other alterations to the opening at the entrance to the pocket.

Structures for new 2-indole carboxamide ligand complexes for GX have also recently become available as a result of a lead generation program dedicated to finding inhibitors selective for GX at AstraZeneca [179,180] (PDB ID 5OWC, 1.75 Å resolution; PDB ID 5OW8, 1.90 Å resolution) [179,180]. Through the discovery of a lead 2-indole compound that was only offering micromolar inhibitory capacity, it represented a relatively low and ideal molecular weight compound with which to begin further rounds of improvement. In particular, the next compounds were found in approaches directed to further exploit the subtype knowledge of the GIIA and V PLA_2_s. Ultimately, a different indole was found, with trifluoromethyloxy added at the indole-6 location, but also attaining nanomolar inhibition (compound 31) [179]. This compound binds differently from other known indole-based inhibitors of PLA_2_s, and so, while the indole rings are actually positioned differently upon binding, the inhibitors are in the same region. A further improvement was reported to be stimulated by introducing polar moieties into the compounds to achieve a better pharmacokinetic profile, and resulted in a structure being published which was the phenyl version from among the heterocycle analogues studied, and with a methyl in the position relative to the carboxylic acid of its attached sidechain (compound S-12) (PDB ID 6G5J, 1.85 Å resolution). Compound 17 had the pyridine ring that was most ideal for use in mouse trials [180], but it unfortunately did not affect lipid and lipoprotein biomarkers in the mouse trials and had no effect on coronary function or histological markers of atherosclerosis. The binding of compounds 31 and S-12 in the structures is very similar, but a shift in the phenyl ring is observable. One can argue that hGIIA remains the best target of the four enzymes inhibited by varespladib, and while the study using compound 17 in mice should be extended, for instance, further dissection of the connection between these enzymes and atherosclerosis is definitely required.

## 10. Summary

In the situation of prostate cancer hGIIA concentrations in serum, prostate tissue and seminal plasma in patients are significantly increased [90,98,99], though, importantly, not all prostate cancer cells express hGIIA. This makes the enzyme a useful biomarker for specific diagnostic purposes, but also underlines it as an important therapeutic target for prostate cancer, as hGIIA also promotes the proliferation of the cancer cells, and its expression level has been observed to be closely associated with the severity of the disease [87]. The pathological role of hGIIA has been described in many diseases, as well as that the specific proinflammatory effects arise through a catalysis-independent mechanism occurring through interaction with other proteins. Out of the several classes of inhibitors targeting hGIIA that have been discovered and developed, the cyclic pentapeptides, cF and c2, derived from the linear peptide FLSYK, displayed selective inhibitory effects towards the catalysis-independent mechanism of hGIIA [13]. Clinical trials targeting the advanced phases of prostate cancer were initiated in 2018 for the most potent compound known in this class, c2, and will become known soon. Yet-to-be delivered inhibitors that are selective will be important for successfully understanding the interplay between the small PLA_2_s in their biological roles, and have the potential to assist in the treatment of many diseases if they can be developed into drugs.

It is encumbent on the field to discern the details of the consequences of selective inhibition of either catalysis alone or the catalysis-independent functions of hGIIA and the closely related and important PLA_2_s. Such studies may lead directly to the development of drugs targeting the specific actions of hGIIA that would potentially and importantly have a better profile than the non-selective inhibitors, as well as drugs specifically targeting other PLA_2_s if and when required.

A role for cytokines is also highly likely in the mechanism for the selective inhibitory effects of the catalysis-independent mechanism of hGIIA, and the roles they play may be important in characteristic behaviours in subsets of cancers, and also be different at different stages of the cancers. An understanding of the roles individual cytokines play in conjunction with hGIIA would be highly enlightening. Out of the several classes of inhibitors targeting hGIIA that have been discovered and developed, the cyclic pentapeptides now hold the promise of delivering new therapies.

## Figures and Tables

**Table 1:** Classification of secretory phospholipases A_2._

Official Name	Alternate Names	Disulfides (Number)	Molecular Mass (kDA)	Catalytic Amino Acids
PLA2G1B	sPLA2–1B, G1B PLA2, pancreatic PLA2	7	13–15	His/Asp
PLA2G2A	sPLA2-IIA, GIIA PLA2	7	13–15	His/Asp
PLA2G2C	sPLA2-IIC, GIIC PLA2	8	15	His/Asp
PLA2G2D	sPLA2-IID, GIID PLA2	7	14–15	His/Asp
PLA2G2E	sPLA2-IIE, GIIE PLA2	7	14–15	His/Asp
PLA2G2F	sPLA2-IIF, GIIF PLA2	7	16–17	His/Asp
PLA2G3	sPLA2-III, GIII PLA2	5	Lizard/Bee: 15–18 Human/Murine: 55	His/Asp
PLA2G5	sPLA2-V, GV PLA2	6	14	His/Asp
PLA2G10	sPLA2-X, GX PLA2	8	14	His/Asp
PLA2G12A	sPLA2-XIIA, GXIIA PLA2	7	19	His/Asp
PLA2G12B	sPLA2-XIIB, GXIIB PLA2	7	19	Leucine/Asp

This table has been adapted from Schaloske and Dennis, 2006 [2] and Murakami and Lambeau, 2019 [3].

**Table 2:** Role of hGIIA in some cancers. Adapted from Brglez et al. 2014 [91].

Cancer	Role	Effects In Vitro	Patient Outcome	Supporting Reference
Breast	Pro-tumorigenic	Unknown	Shorter patient survival	Brglez et al. 2014 [92]
Colon	Pro/Anti-tumorigenic	Increased cell proliferation	Unknown	Avoranta et al. 2010 [93]
Gastric	Anti-tumorigenic	Reduction in cell migration and invasiveness	Longer patient survival, less frequent metastasis	Wang et al. 2013 [94]
Lung	Pro-tumorigenic	Increased cell proliferation, lower rates of apoptosis	Shorter patient survival	Yu et al. 2012 [95]
Oesophageal	Pro-tumorigenic	Increased cell proliferation	Unknown	Menschikowski et al. 2013 [96]
Prostate	Pro-tumorigenic	Increased cell proliferation	Shorter patient survival	Oleksowicz et al. 2012 [97]

Also see the extensive referencing in Table 1 of Brglez et al. 2014 [91].
